# Neuralgia of Dental Origin.—Its Discrimination and Treatment

**Published:** 1884-07

**Authors:** J. Morley Dennis

**Affiliations:** Dental Surgeon to the Grimsby and District Hospital.


ARTICLE V.

### OBSERVATIONS ON NEURALGIA OF DENTAL ORIGIN; ITS DISCRIMINATION AND OPERATIVE TREATMENT.

BY J. MORLEY DENNIS,

(Dental Surgeon to the Grimsby and District Hospital.)

Of all the secondary affections connected with the teeth none is more troublesome than Neuralgia. It may be described as a disease of the nervous system manifesting itself by pains, which, in most cases, are unilateral, and which appear to follow accurately the course of particular nerves, and ramify, sometimes into a few, and sometimes into all of their terminal branches. It occurs in paroxysms, perhaps at nearly equidistant intervals, the pains being usually sudden in their onset, and of a stabbing, boring or burning character. The attack increases in severity on each successive occasion, while between the paroxysms the patient is usually free from pain, and the attack often ceases as suddenly as it comes on. It has been said that from a pathological point of view the disease Neuralgia probably has no existence; it is but a symptom indicative of a lesion at some points, which may be discoverable, or may be hidden from our view; and it is not indicative of any one particular lesion, but of a great variety of morbid conditions. It often consists of pain, and of nothing else that we can perceive, and in many cases is attended by no inflammation, no detectable change of structure, and no fever. That pain is owing to some morbid condition, or to some irritation of a particular nerve, we may sometimes know by finding that it is felt exactly in the course, and following the distribution of that particular nerve; but in the abscence of any such symptoms we have some difficulty in forming a correct diagnosis; and again the difficulty is increased when we consider that it may be produced by some source of irritation operating at a distance from the part in which the pain is felt. It may be in the brain itself, or in the spinal cord; in the trunk of the nerve that supplies the effected part, or in one of the remote branches of the same trunk, so that it is necessary when we can find no explanation of a pain in the place in which it is felt to look for some condition that may explain it in the trunk of the nerve supplying that part, or in the parts supplied by other branches of the same nerve. These remarks refer more particularly to Neuralgia of the trifacial nerve, which from the similarity of of its symptoms to those of other diseases affecting the teeth —the dental pulp, and the alveolar periosteum—is liable to to be mistaken for one or other of them, as also Neuralgia of dental origin is often mistaken for Neuralgia arising from other causes. It is, therefore, not only necessary that we should make ourselves acquainted with the symptoms of each, in order that in case it arises from irritation or any other morbid condition of the teeth or alveolus, we may, if possible, remove that irritation, and, on the other hand, if it arises from other causes, we may at once refer it to the general surgeon for treatment, but that we should also be able to define the locality of that irritation.

In making an examination of patients, with a view to the discovery of the pain from which they are suffering—and I cannot but consider this description of neuralgia rather as a symptom than as a disease *per se*, because, from whatever cause it arises, it is the effect of some condition existing sometimes at a considerable distance—we must not, however, place much reliance on the statements of patients, without at the same time giving due consideration to the possibility of the pain being reflected, and thus misleading us as to its origin. Many cases have been related in which the patient has ascribed pain to be due to some particular tooth, and which, on fuller investigation, proved to be caused by irritation from another tooth existing in a different part of the mouth, and in some cases the antagonistic tooth of the opposite jaw. Mr. Tomes relates a case in which this perverted sensation was so definite that it was with some difficulty he persuaded the patient that the source of his trouble was not where he felt the pain.

A gentlemen requested him to extract a perfectly sound second *upper* molar, the tooth affected being the corresponding *lower* molar of which the pulp was exposed. The *lower* tooth was extracted under nitrous oxide, but so soon as he recovered he exclaimed, "You have taken out the *upper* tooth after all;" nor could he be persuaded, until he felt the vacant space with his fingers, that such was not the case.

Mr. Stewart some time ago brought before the notice of the profession another subject which it will repay us to consider at the present time; he points out clearly the misleading effects of attention in cases of facial neuralgia from dental irritation, which, he says, "Must be continually kept in view in the practice of Dental Surgery, otherwise disappointment in the results of operations would be frequent. In a large proportion of cases of Neuralgia dependent on dental irritation the patient has a foregone conclusion that a tooth which he indicates is the source of the pain—a conclusion so firmly held that he has resolved on the extraction of the tooth, and is astonished and incredulous when told, after examination, that not it, but another, is really the seat of the pain. It is often difficult, and sometimes impossible, to compel belief that a tooth he had no reason to suspect, probably in the opposing jaw and several teeth distant, is the cause of all he has suffered, and should be extracted rather than the one he was prepared to lose. As cavities that produce acute dental irritation are generally recent, sometimes cause no pain in the tooth, and are often such as cannot possible be seen or felt by the patient, he is obliged to search for source of pain, and any tooth on the same side with the pain, that presents some altered appearance, is fixed on, and immediately, somehow or other, has pain transmitted to it. A dark stain merely, a chip—probably an old one—a root, however small, and especially a cavity plainly seen or felt, suffices to direct attention and pain to a particular tooth, although the stained part be sound, the chipped surface harmless, the root incapable of mischief, and the cavity dormant." It will be observed, therefore, how easily we may be mistaken in our diagnosis, unless we make a careful examination of the entire mouth with a determined intention to come at the true cause of the mischief, rather than lose the confidence of our patient by sacrificing a useful tooth without affording him any relief, for the sake of avoiding a little trouble. One difficulty we have to meet is that patients will not always act upon the advice given; in this case, however, they must take the consequences.

Neuralgia may arise from chronic inflammation of the dental pulp, difficult eruption of wisdom teeth, secondary dentine in the pulp cavity ; decomposition of a dead pulp in a confined space; exostosis; alveolar periostitis, which may depend on the escape of decomposition matter through the pulp canal, or on roughening of the fang by absorption, exposure of sensitive dentine, etc., the most common cause being chronic inflammation of the pulp. Neuralgia may attack any of the three divisions of the fifth nerve, but rarely more than one at a time, and generally either the second or third. M Trosseau attaches great importance to the diagnosis value of the tenderness sometimes felt over the first two cervical vetebræ ; in this, however, Dr. Anstie and Mr. Tomes do not entirely agree. The intermittent character of the pain is regarded by some as of great value in the recognition of the disease. If the pain is most acute about the temple, the irritating tooth is generally situated far back in the upper jaw ; when it is referred to the condyles the offending organ is generally in the back part of the lower jaw. These rules will aid us when it is caused by the difficult eruption of wisdom teeth, which should be cut down upon and removed, and if impaction is suspected the second molar may be extracted. When the pain is due to exostosis, absorption of the fang or secondary dentine extraction is necessary. In chronic inflammation of the pulp, if possible, treat the pulp antiseptically, cap and fill ; if not, it must be destroyed and removed. In cases arising from decomposition of dead pulp, open out cavity, clear away decomposed matter, and disinfect. When alveolar periostitis exists try leeches, iodine, aconite, etc.—*London Dental Record.*

